# 2455. National Trends in the Rates of Drug-resistant Bacteria Commonly Associated with Healthcare in U.S. Acute Care Hospitals, 2019-2021

**DOI:** 10.1093/ofid/ofad500.2073

**Published:** 2023-11-27

**Authors:** James Baggs, Hannah Wolford, Natalie McCarthy, Babatunde Olubajo, Deana Li, Alex Maillis, Sujan Reddy

**Affiliations:** CDC, atlanta, Georgia; CDC, atlanta, Georgia; CDC, atlanta, Georgia; CDC, atlanta, Georgia; CDC, atlanta, Georgia; CDC, atlanta, Georgia; CDC, atlanta, Georgia

## Abstract

**Background:**

The *2022 Special Report: COVID-19 U.S. Impact on Antimicrobial Resistance* identified significant increases in the rate of hospital-onset (HO) infections during the year 2020 for bacteria commonly associated with healthcare. Using the same data sources and methodology, we provide updated estimates for the year 2021.

**Methods:**

We identified a cohort of patients from the PINC AI PHD and BD Research Insights databases with a clinical culture yielding an isolate of an organism of interest with accompanying susceptibility testing. Isolates from patients with no culture yielding the same resistance phenotype of interest in the previous 14 days were counted as an incident case. Community-onset (CO) cultures were obtained ≤ day 3 of hospitalization; HO cultures were obtained ≥ day 4. We used a raking-procedure to determine weights for extrapolating the number of discharges included in our sample to match the distribution of discharges, stratified by bed size, U.S. census division, urban/rural designation, and teaching status, for U.S. hospitals included in the American Hospital Association survey. We examined temporal trends using a multivariable logistic model incorporating a survey design with the corresponding weights and hospital designation as the specific cluster controlling for hospital characteristics, month of discharge, proportion of patients in specific age group, proportion of male patients, and database. Results were stratified by HO and CO.

**Results:**

From 2019-2021, the overall number of hospitals contributing data was 640. HO rates were significantly higher in 2020 and 2021 compared with 2019. The HO rates of VRE, MDRPseu, and CRE were significantly higher in 2021 when compared to 2020 (Table 1). For CO infections, we found that MRSA rates decreased significantly during the study period, while VRE and CRAB were significantly higher in 2021 compared to 2019 (Table 1).

**Figure:**
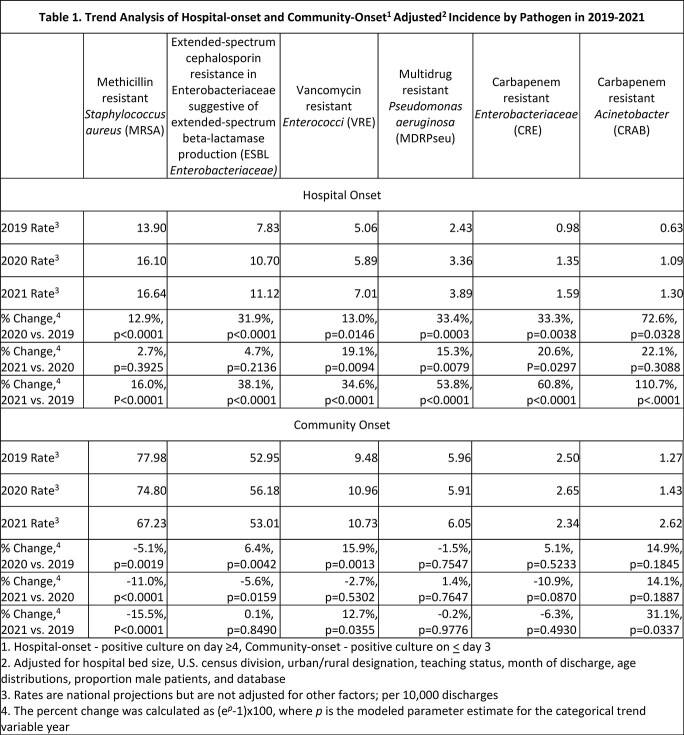

**Conclusion:**

Hospital onset antibiotic resistant infection rates in 2021 remain higher than pre-pandemic rates with increasing rates for several pathogens in 2021 compared to 2020, while CO MRSA declined. As infections caused by resistant pathogens represent a serious threat to patient safety, further study of factors contributing to the continued increases and decreases may inform prevention strategies.

**Disclosures:**

**All Authors**: No reported disclosures

